# Senotherapeutics: An emerging approach to the treatment of viral infectious diseases in the elderly

**DOI:** 10.3389/fcimb.2023.1098712

**Published:** 2023-03-29

**Authors:** Zhiqiang Li, Mingfu Tian, Guolei Wang, Xianghua Cui, Jun’e Ma, Siyu Liu, Bingzheng Shen, Fang Liu, Kailang Wu, Xuan Xiao, Chengliang Zhu

**Affiliations:** ^1^ Department of Clinical Laboratory, Institute of Translational Medicine, Renmin Hospital of Wuhan University, Wuhan, China; ^2^ State Key Laboratory of Virology, College of Life Sciences, Wuhan University, Wuhan, China; ^3^ Department of Pharmacy, Renmin Hospital of Wuhan University, Wuhan, China

**Keywords:** senescence, virus, COVID-19, cGAS-STING, NLRP3 inflammasome, senotherapeutics

## Abstract

In the context of the global COVID-19 pandemic, the phenomenon that the elderly have higher morbidity and mortality is of great concern. Existing evidence suggests that senescence and viral infection interact with each other. Viral infection can lead to the aggravation of senescence through multiple pathways, while virus-induced senescence combined with existing senescence in the elderly aggravates the severity of viral infections and promotes excessive age-related inflammation and multiple organ damage or dysfunction, ultimately resulting in higher mortality. The underlying mechanisms may involve mitochondrial dysfunction, abnormal activation of the cGAS-STING pathway and NLRP3 inflammasome, the role of pre-activated macrophages and over-recruited immune cells, and accumulation of immune cells with trained immunity. Thus, senescence-targeted drugs were shown to have positive effects on the treatment of viral infectious diseases in the elderly, which has received great attention and extensive research. Therefore, this review focused on the relationship between senescence and viral infection, as well as the significance of senotherapeutics for the treatment of viral infectious diseases.

## 1 Introduction

Although the increasing aging population worldwide indicates that the average life expectancy of humans has lengthened, a simultaneous increase in age-related chronic diseases has also been observed. Simultaneously, there has been unprecedented interest in aging-related research, especially during the global COVID-19 pandemic, in which the elderly were found to suffer from higher morbidity and mortality compared to other age groups (Grasselli et al., 2020; Onder et al., 2020).

Age is considered a critical risk for the severity of COVID-19 disease (Zhou et al., 2020; Wang et al., 2020a). Data from China showed that the case fatality rate (CFR) of COVID-19 increased with age, and the CFR for patients aged 40 years or younger was ≤0.4%, but rose to 8.0% in patients aged 70 to 79 years, and 14.8% in patients aged ≥80 years (The Novel Coronavirus Pneumonia Emergency Response Epidemiology Team, 2020). Similarly, data from Italy revealed that CFR was also ≤0.4% for patients aged ≤40 years and, rose to 12.8% and 20.2% in patients aged 70 - 79 years and ≥80 years, respectively (Onder et al., 2020). In addition, a retrospective study on 5256 COVID-19 patients in the United States found that old age, male sex and impaired physical or cognitive function were independent risk factors for 30-day mortality (Panagiotou et al., 2021). Overall, current epidemiological evidence suggests that elderly COVID-19 patients (age ≥80 years) have a significantly higher risk of death than younger patients (Ackermann et al., 2020; Akbar and Gilroy, 2020). Moreover, higher mortality rates have also been reported in the elderly with influenza virus and respiratory syncytial virus infections (Thompson et al., 2003).

Thus, there are several unanswered questions between viral infections and senescence, such as: why do older people have higher morbidity and mortality from viral infections or how do viral infections and senescence interact and influence each other? To answer these questions, this review focuses on the relationship between senescence and viral infections and discusses the significance of senescence-targeted drugs for the treatment of viral infectious diseases, so as to provide insights for better understanding the role of senescence in disease development.

## 2 Characteristics of senescence

A basic feature of aged organisms is the accumulation of senescent cells. Senescence is a permanent status of cell cycle arrest in normal proliferating cells, described back in the 1960s when Hayflick and Moorhead found that the proliferation ability of cultured human diploid cells was limited and that cells stopped proliferating after serial passage *in vitro* (Hayflick and Moorhead, 1961; Hayflick, 1965; Lynch et al., 2021). Since then, biologists have gained a more comprehensive understanding on the characteristics associated with senescence.

### 2.1 Inducing factors of senescence

**Figure 1:**
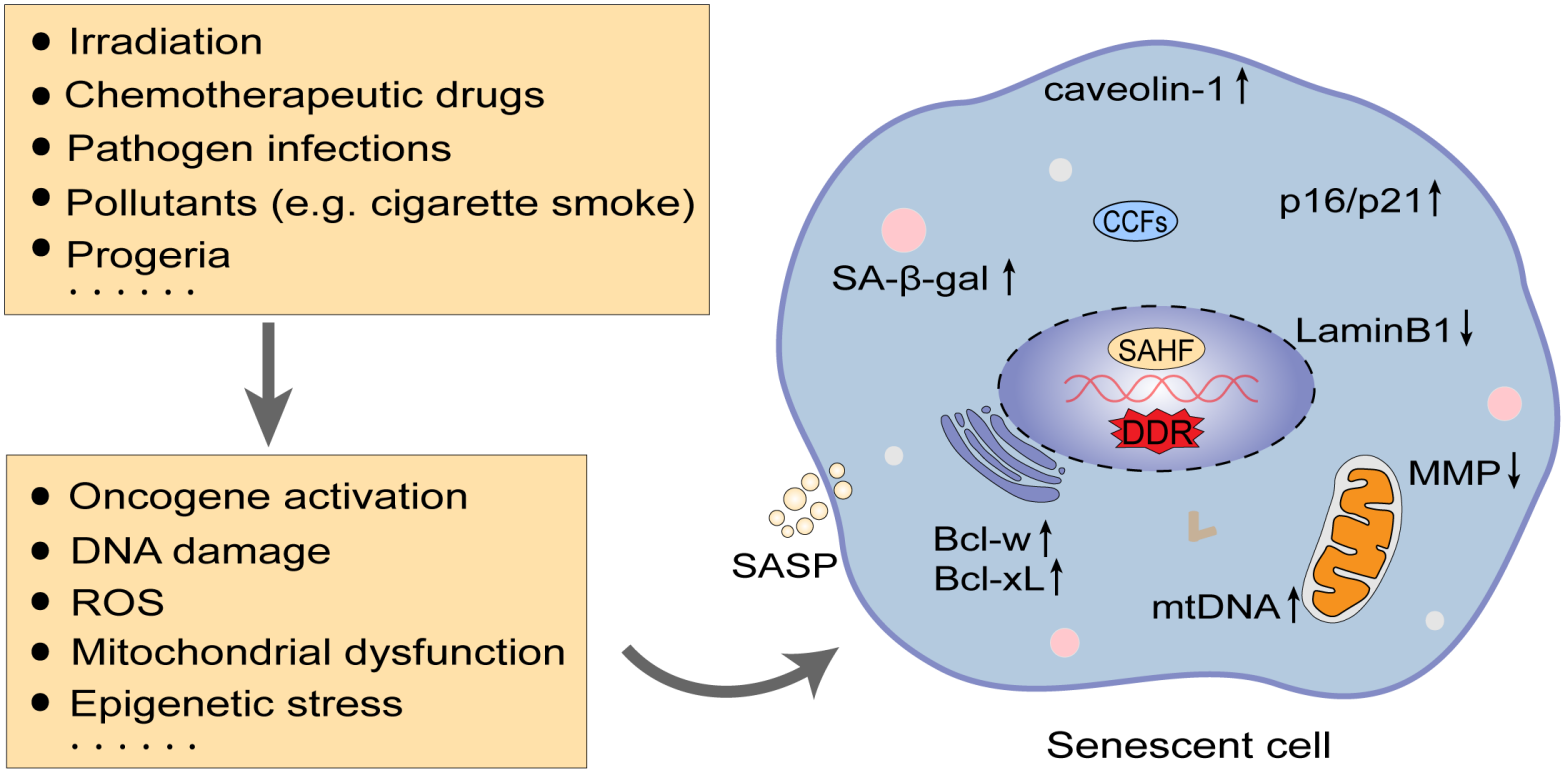
Inducing factors and characteristics of senescence. In addition to replicative senescence induced by telomere shortening, numerous factors such as irradiation, chemotherapeutic drugs, and pathogenic infections can evoke senescence by impacting oncogene activation, DNA damage, mitochondrial dysfunction, etc. DNA damage response (DDR) is an essential mechanism in triggering senescence. Cellular senescence features altered cell morphology and structure, arrest of the cell cycle (upregulation of p16/p21), and enhanced resistance to apoptosis (upregulation of Bcl-xL and Bcl-w), along with detectable nuclear senescence-associated heterochromatin foci (SAHFs), increased senescence-associated β-galactosidase (SA-β-gal) activity, and secretion of senescence-associated secretory phenotype (SASP). ROS, reactive oxygen species; MMP, membrane potential; CCFs, cytoplasmic chromatin fragments; mtDNA, mitochondrial DNA.

Cellular senescence is caused by repeated cell divisions and cellular stressors (Kelley et al., 2020). Replicative senescence results from repeated cellular divisions and has been confirmed to be related to the gradual shortening of telomeres during cell division (Shay, 2016; Lynch et al., 2021). Stress-induced senescence arises from cellular stressors, such as oncogene activation, DNA damage, reactive oxygen species (ROS), mitochondrial dysfunction and epigenetic stress (Hernandez-Segura et al., 2018), which may result from irradiation, chemotherapeutic drugs, pathogen infections, long-term exposure to pollutants (cigarette smoke) and certain aging syndromes such as progeria (Nyunoya et al., 2006; Nyunoya et al., 2009; Wheaton et al., 2017; Kelley et al., 2020; Di Micco et al., 2021) (**Figure 1**). Therefore, cells from both young and aged hosts may exhibit senescent properties (Kelley et al., 2020).

Activation of DNA damage response (DDR) signaling cascades initiated by nucleus DNA double-strand breaks (DSBs) is considered to be a common factor to induce cellular senescence (Di Micco et al., 2021). Specifically, there are two kinases upstream of DDR, known as ataxia telangiectasia mutated (ATM) and ATM- and Rad3-Related (ATR) protein kinases, they are respectively activated by the MRE11-RAD50-NBS1 (MRN) complex at DSBs and the TopBP1 or ETAA1 at replication protein A coated ssDNA (RPA-ssDNA). Activated ATR and ATM can further phosphorylate the downstream kinases CHK1 and CHK2, respectively, which in turn activate the p53 pathway, leading to cell cycle arrest (Blackford and Jackson, 2017; Di Micco et al., 2021). Importantly, factors that can cause DNA damage, such as telomere shortening, oncogene activation and ROS, would ultimately be involved in activating the DDR pathway(van Deursen, 2014). If DNA damage from various causes persists, prolonged DDR signaling and proliferation arrest can invoke the onset of cellular senescence (Fumagalli et al., 2014). In addition, studies have reported that IFN-β secreted by senescent cells can stimulate DDR through ROS and generate senescence-like cell cycle arrest in human fibroblasts, which can trigger positive feedback activation of DDR and further amplify the senescence phenotype (Yu et al., 2015).

### 2.2 Hallmarks of senescence

Senescent cells have various characteristics (Hernandez-Segura et al., 2018) (**Figure 1**): (1) Morphologically, senescent cells are abnormally enlarged and flattened, with a disproportionate increase in the cytoplasm and nuclei (Bent et al., 2016; Druelle et al., 2016; Cormenier et al., 2018; Di Micco et al., 2021); changes in the composition of the plasma membrane, such as caveolin-1 protein upregulation (Dasari et al., 2006; Chrétien et al., 2008; Althubiti et al., 2014); increased lysosome content and some proteins (Cho and Hwang, 2012); accumulated mitochondria and decreased membrane potential (MMP) (Passos et al., 2007; Korolchuk et al., 2017; Tai et al., 2017); nuclear membrane structural protein loss, such as the downregulation of LaminB1 protein and presence of nuclear senescence-associated heterochromatin foci (SAHFs) with detectable dense 4’,6-diamidino-2-phenylindole (DAPI)-positive nuclear structural features (Di Micco et al., 2011; Sadaie et al., 2013); (2) Elevated senescence-associated β-galactosidase (SA-β-gal) activity: SA-β-gal is a lysosomal-derived enzyme that is regarded as a surrogate marker for increased lysosomal content in senescent cells and is one of the most common markers of senescence (Dimri et al., 1995; Kurz et al., 2000; Lee et al., 2006); (3) Accumulation of cyclin-dependent kinase inhibitors (CDKis): Senescence-related cell cycle arrest is primarily driven by CDKis encoded at *CDKN2A* (p16^INK4a^ or p16), *CDKN2B* (p15^INK4b^ or p15) and *CDKN1A* (p21^CIP1^ or p21) loci. p21 and p16 maintain the tumor suppressor protein pRb in an inactive hypophosphorylated state, thereby preventing the transcription factor E2F from transcribing genes that promote cell cycle progression, and both are often used as unique senescence hallmarks to identify senescent cells in tissues and cultured cells (Narita et al., 2003; Baker et al., 2011; Di Micco et al., 2021); (4) Senescence-associated secretory phenotype (SASP): SASP consists of various cytokines, chemokines and some enzymes involved in extracellular matrix remodeling, mainly including IL-1α/β, IL-6, IL-8, TNF-α, TGF-β, monocyte chemotactic protein 1 (MCP1, also known as CCL-2) and matrix metalloproteinases (MMPs). SASP is thought to be the main mechanism by which senescent cells exert their pleiotropic biological functions and can also induce paracrine senescence (Freund et al., 2010; Acosta et al., 2013; Tchkonia et al., 2013; Gorgoulis et al., 2019); (5) Enhanced apoptosis resistance: Senescent cells stimulate a wide range of pro-survival factors, such as BCL-2 family members, particularly Bcl-xL and Bcl-w, which can be resistant to apoptosis and favor the survival of senescent cells (Childs et al., 2014; Yosef et al., 2016).

Defects associated with aging of the immune system are another feature of aging, termed “immunosenescence” (Kelley et al., 2020). It is characterized by decreased proliferation of hematopoietic stem cells, dysfunction of innate immunity, degeneration of the thymus and reduced numbers of naïve T and B cells, as well as accumulation of memory T and B cells, and decline in T and B cell functions (Kelley et al., 2020; Xu et al., 2020). Immunosenescence is associated with increased susceptibility to various diseases, such as infections, cancer, cardiovascular diseases, hypertension, diabetes, neurological dysfunction, and autoimmune diseases (Xu et al., 2020).

## 3 Virus-induced senescence

Virus infections can prematurely stimulate cellular senescence, known as virus-induced senescence (VIS). Studies have shown that some viruses, such as the human immunodeficiency virus (HIV), measles virus (MV), respiratory syncytial virus (RSV) and influenza virus, can induce cell fusion and form multinucleated cells upon infecting the organism as a mechanism for expanding its spread in the infected organisms (Chen and Olson, 2005; Duelli and Lazebnik, 2007; Sapir et al., 2008; Delpeut et al., 2012). MV infection has been proven to induce p53 and p16-pRb pathway-dependent cellular senescence *via* cell fusion (Chuprin et al., 2013). Epstein–Barr virus (EBV), Kaposi sarcoma herpesvirus (KSHV) and human RSV infections can trigger DNA damage-mediated cellular senescence through replicative stress or induction of mitochondrial ROS (Koopal et al., 2007; Martínez et al., 2016; Hafez and Luftig, 2017). Some viral proteins, such as NS1 of influenza A virus (IAV) (Yan et al., 2017), HBx of hepatitis B virus (HBV) (Idrissi et al., 2016) and IE2 of human cytomegalovirus (CMV) (Noris et al., 2002), were found to induce senescence by increasing the inducible NO synthase (iNOS) expression and NO release and regulating the p21 and p16 pathways, respectively. HIV Tat and Nef proteins can provoke bone marrow mesenchymal stem cells senescence through either enhanced inflammation or reduced autophagy (Beaupere et al., 2015), and HIV Tat can also trigger microglia senescence upon miR-505-SIRT3 axis-mediated mitochondrial oxidative stress (Thangaraj et al., 2021) (**Table 1**).

**Table 1:** Virus-induced senescence and potential mechanism.

Virus	Mechanism	Refs
Measles virus	Cell fusion and induction of p53 and p16-pRb pathways	(Chuprin et al., 2013)
Respiratory syncytial virus	Mitochondrial ROS production and DNA damage response	(Martínez et al., 2016)
Kaposi sarcoma herpesvirus	Oncogene activation and DNA damage response	(Koopal et al., 2007)
Epstein-Barr virus	Replicative stress and DNA damage response	(Hafez and Luftig, 2017)
Influenza A virus	NS1 protein increases the iNOS expression and NO release; SASP-related paracrine senescence	(Yan et al., 2017; Lv et al., 2022; Schmitt et al., 2022)
Hepatitis B virus	HBx C-terminal mutants of HBV regulate the p21 and p16 pathways	(Idrissi et al., 2016)
Cytomegalovirus	IE2 protein regulates the p53 and p16 pathways	(Noris et al., 2002)
Human immunodeficiency virus	Induction of immunosenescence; HIV Tat protein augments miR-505-SIRT3 axis-mediated mitochondrial oxidative stress and enhances inflammation; HIV Nef protein reduces autophagy	(Beaupere et al., 2015; Blanco et al., 2021; Thangaraj et al., 2021; Chauvin and Sauce, 2022)
SARS-CoV-2	Activation of DNA damage response; SASP-related paracrine senescence	(Lee et al., 2021; Evangelou et al., 2022; Lv et al., 2022; Schmitt et al., 2022)
Herpes simplex virus 1	Activation of p53/p16 pathways and NLRP3	(Sivasubramanian et al., 2022)

ROS, reactive oxygen species; iNOS, inducible nitric oxide synthase; NO, nitric oxide.

The occurrence of VIS was assessed in a basic research study (Lee et al., 2021), which found that human diploid fibroblast models exposed to high-titer retrovirus exhibited typical characteristics of senescence and the activated cyclic GMP-AMP synthase-stimulator of interferon genes (cGAS-STING) pathway after the fifth day of infection. Consistently, VIS was detectable in human lung carcinoma cells and non-malignant epithelial cells upon infection with lentivirus, adeno-associated virus (AAV), vesicular stomatitis virus (VSV) and the low-pathogenic human alphacoronavirus NL63 (HCoV-NL63). In parallel, canonical cellular senescence phenotype were found in SARS-CoV-2-infected human primary nasal epithelial cells (HNEpc), alveolar epithelial cells (AEC), normal human bronchus epithelial (NHBE) cells and macrophages, and COVID-19 patients also displayed marked signs of senescence in their nasopharyngeal and lung tissue specimens and elevated serum levels of SASP factors, suggesting that SASP-mediated effects are pivotal factors in secondary paracrine senescence, lung disease, hyperinflammation, tissue damage, and coagulation disorders of patients infected with SARS-CoV-2 (Wiley et al., 2019; Ackermann et al., 2020; Lee et al., 2021; Evangelou et al., 2022; Schmitt et al., 2022). Likewise, a recent study (Lv et al., 2022) also indicated that aged mice deficient in telomerase RNA (Tere^-/-^) were extremely sensitive to IAV, SARS-CoV-2 and other respiratory virus infections. Tere^-/-^ mice showed typical features of cellular senescence and aberrant activation of the cGAS-STING pathway and NOD-like receptor family pyrin domain containing 3 (NLRP3) inflammasome mediated by leaked mitochondrial DNA (mtDNA), which could contribute to an excessive inflammatory response, particularly following viral exposure, thereby were more likely to develop severe viral pneumonia in non-fatal respiratory virus infections and abnormally increased mortality for Tere^-/-^ mice (Ackermann et al., 2020; Akbar and Gilroy, 2020; Decout et al., 2021; Sanchez-Vazquez et al., 2021; Xian et al., 2021; Lv et al., 2022). Further, latent herpes simplex virus 1 (HSV-1) infection in the key brainstem regions of female mice induces senescence by activating the p53/p16 pathway and NLRP3, resulting in neuroinflammation and neurodegeneration (Sivasubramanian et al., 2022).

HIV infection can also induce a senescent phenotype with the same characteristics as normal senescence (Appay et al., 2007). Owing to antiretroviral therapy (ART), the life expectancy of HIV-infected persons (PLWH) has increased (Wandeler et al., 2016). However, there is still persistent immune activation and inflammation in PLWH even though the virus is effectively suppressed, thus contributing to premature aging (Blanco et al., 2021; Chauvin and Sauce, 2022).

Acquired immune deficiency syndrome (AIDS) patients are often co-infected with the herpes virus (CMV, EBV and HSV), HBV and hepatitis C virus (HCV), of which CMV is the most common chronic infection (Blanco et al., 2021). Chronic CMV infection is highly prevalent in the HIV-negative general elderly population and nearly universal in the HIV-positive elderly population, enabling T-cell clonal expansion and leading to immunosenescence and chronic low-grade inflammation (Khan et al., 2002; Koch et al., 2007; Leng and Margolick, 2020), whereas CMV and HIV co-infection can cause further adverse effects (Leng and Margolick, 2020). Interestingly, AIDS patients have reduced levels of Kupffer cells and CD4^+^ T cells in the presence of HIV and HCV co-infection, which can lead to a decrease in the clearance of microbial products and an increase in the levels of soluble CD14 (sCD14), lipopolysaccharide (LPS), peptidoglycan, and ribosomal DNA in the blood *via* microbial translocation (Ancuta et al., 2008; Sandler and Douek, 2012; Blanco et al., 2021). These microbial products can bind to pattern recognition receptors (PRRs) and trigger signaling cascades that favor chronic immune activation and inflammation (Sandler and Douek, 2012; Vázquez-Castellanos et al., 2015; Dillon et al., 2016). At the same time, a reduction in the number of CD4^+^ T cells during HIV infection may promote the replication of HCV, resulting in CD8^+^ T cells being continuously activated. This reciprocates the cycle of viral replication and immune activation, showing signs of activation, exhaustion, and immunosenescence (Appay and Kelleher, 2016; Hoffmann et al., 2016; Blanco et al., 2021).

PLWH has similar features as natural immunosenescence. For instance, PLWH is associated with decreased numbers and impaired proliferative capacity of circulating CD34^+^ hematopoietic progenitor cells (HPCs), thymic degeneration, reduced initial T cells and an accumulation of memory T cells. They also have reduced CD56^++^NK cells and CD14^++^CD16^-^classical monocytes while increased CD14^++^ CD16^+^ intermediate and CD14^+^ CD16^++^ non-classical monocytes (Hakim et al., 2005; Seidler et al., 2010; Sauce et al., 2011; Hearps et al., 2012; Naranbhai et al., 2013; Massanella et al., 2015; Chauvin and Sauce, 2022). These factors can increase the risk of various age-related diseases in PLWH, such as cardiovascular diseases, renal failure, liver diseases, osteoporosis, cancer and cognitive dysfunctions (Deeks et al., 2013; Gallant et al., 2017).

## 4 Consequences of senescence

**Figure 2:**
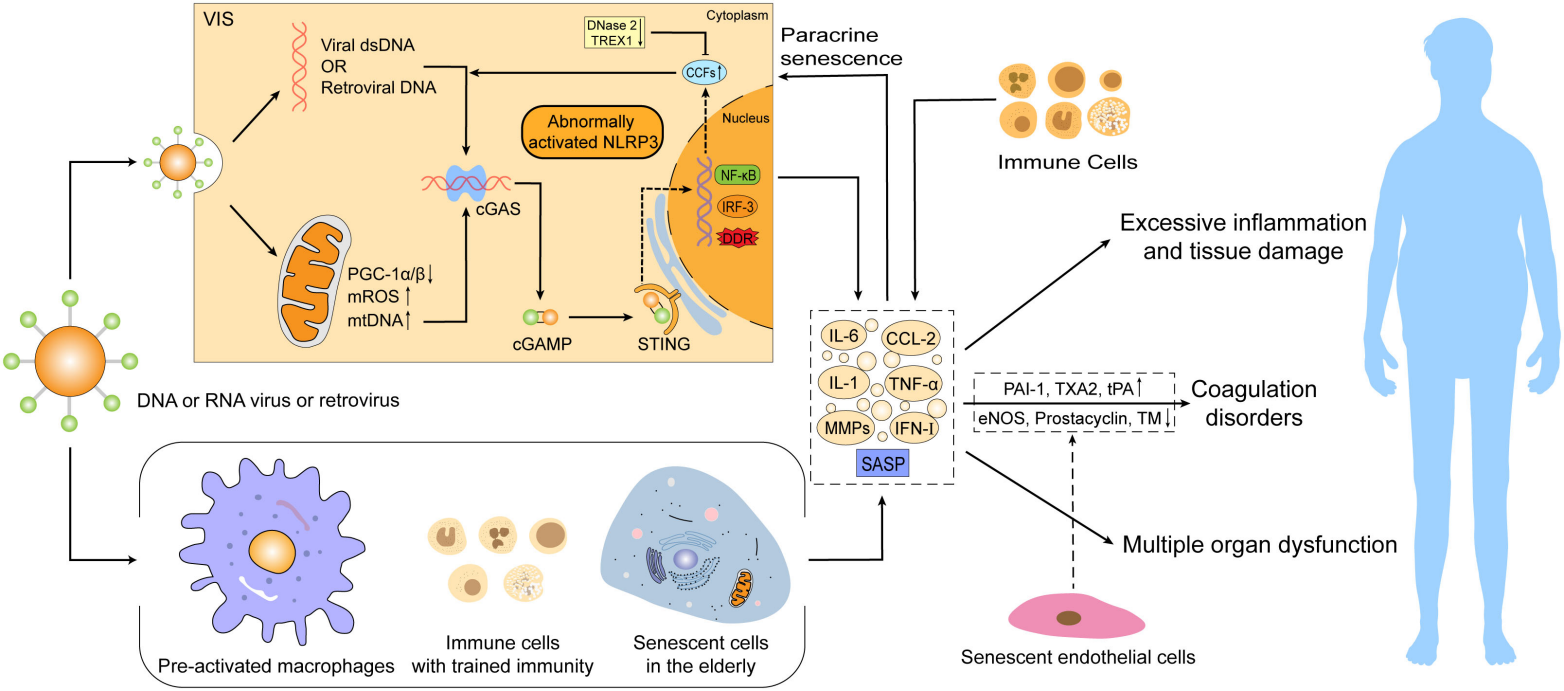
Virus-induced senescence (VIS) combined with an existing senescence in the elderly intensifies the severity of viral infections. Senescence can promote the development of viral infectious diseases *via* abnormal activation of the mtDNA/CCFs-cGAS-STING pathway and NLRP3 inflammasome, pre-activated macrophages, over-recruited immune cells, and accumulation of innate immune cells with “trained immunity” characteristics. These alterations can trigger excessive SASP production and secondary paracrine senescence, resulting in hyperinflammation, tissue damage, coagulation disorders, and even multiple organ dysfunction, thus leading to a higher mortality risk. SASP, senescence-associated secretory phenotype; cGAMP, cyclic GMP–AMP; CCFs, cytoplasmic chromatin fragments; DDR, DNA damage response; eNOS, endothelial NO synthase; PAI-1, plasminogen activator inhibitor-1; TXA2, thromboxane A2; TM, thrombomodulin; ROS, reactive oxygen species.

With an increase in age, organisms tend to turn into a pro-inflammatory state characterized by low levels of circulating pro-inflammatory factors and perpetuate chronic inflammation in the elderly population (Franceschi et al., 2000; Xu et al., 2020). However, in the event of viral infections, virus-induced senescence combined with an existing senescence in aged or vulnerable hosts may trigger a more intense inflammatory cascade response, leading to more severe symptoms and multi-organ damage or even dysfunction in the elderly infected population, and thus a higher mortality rate (**Figure 2**).

### 4.1 Excessive inflammation and tissue damage associated with aging

As aging occurs, cumulative senescent cells accelerate chronic inflammation through senescence-associated secretory phenotype (SASP), whereby SASP demonstrates a double-edged sword role (Hernandez-Segura et al., 2017). On the one hand, short-term SASP secretion promotes tissue repair and wound healing (Jun and Lau, 2010; Demaria et al., 2014) and enhances immune surveillance to inhibit tumor progression and pathological fibrosis (Xue et al., 2007; Krizhanovsky et al., 2008; Kang et al., 2011; Sagiv et al., 2016), while on the other hand, prolonged SASP secretion contributes to the development of age-related chronic inflammatory diseases by triggering over-recruitment of immune cells (Muñoz-Espín and Serrano, 2014; Childs et al., 2015; He and Sharpless, 2017). Excessive SASP can also recruit immature myeloid cells to favor tumorigenesis and tumor progression in a paracrine manner and affect tissue regeneration by limiting the proliferative potential of stem and progenitor cells (Yoshimoto et al., 2013; Di Mitri et al., 2014; Eggert et al., 2016; Gonzalez-Meljem et al., 2017; Di Micco et al., 2021). The mechanisms of excessive inflammation and tissue damage caused by aging may involve the abnormal activation of the mtDNA/CCFs-cGAS-STING pathway and NLRP3 inflammasome, the role of pre-activated macrophages and over-recruited immune cells and the accumulation of innate immune cells with trained immunity (**Figure 2**).

#### 4.1.1 Abnormal activation of the mtDNA/CCFs-cGAS-STING pathway and NLRP3 inflammasome

Aging-related mitochondrial dysfunction is identified as a potential mechanism leading to increased inflammation. Mitochondria are extremely important organelles involved in a wide range of cellular activities, such as oxidative phosphorylation, ATP synthesis, apoptosis, autophagy and immune responses (Nunnari and Suomalainen, 2012; Mills et al., 2017; Lv et al., 2022). Thus, complete mitochondrial structure and function are essential for maintaining cellular homeostasis and physiological function (Nunnari and Suomalainen, 2012). However, the adaptability and integrity of mitochondria gradually deteriorate with age, thereby provoking a decline in oxidative phosphorylation efficiency, decrease in MMP, impairment in ATP generation, increase in ROS production and altered autophagic activity, ultimately prompting the leakage of mtDNA from dysfunctional mitochondria (López-Otín et al., 2013; Korolchuk et al., 2017; Hopfner and Hornung, 2020; Lv et al., 2022). Importantly, mtDNA can be recognized by the cGAS-STING system to trigger immune and inflammatory responses (Hopfner and Hornung, 2020; Lv et al., 2022). Specifically, cGAS can directly bind to DNA released into the cytoplasm and subsequently synthesize cyclic GMP–AMP (cGAMP) from GTP and ATP. STING is bound and activated by cGAMP, which activates the NF-κB and IRF3 pathways, thus inducing the production of type I interferon and pro-inflammatory cytokines such as IL-1 and IL-6 (Hopfner and Hornung, 2020; Yang et al., 2021).

In addition to mitochondrial-derived mtDNA, there is evidence that age-related reduction of the LaminB1 protein compromises nuclear envelope integrity and causes the accumulation of cytoplasmic chromatin fragments (CCFs), which can also activate the cGAS-STING pathway and intensify the production of pro-inflammatory factors (Ivanov et al., 2013; Shah et al., 2013; Dou et al., 2017; Glück et al., 2017). Likewise, increased DNA in the cytoplasm caused by telomere dysfunction can be detected by cGAS (Chen et al., 2017; Nassour et al., 2019). The cumulation of nuclear DNA in the cytoplasm is associated with the downregulation of DNases involved in cytoplasmic DNA degradation in senescent cells, such as DNase 2 and TREX1 (Takahashi et al., 2018). Moreover, senescence-related impairment of autophagy, which delays the clearance of activated STING and other cellular debris, can also lead to further accumulation of cytoplasmic DNA and amplify the cGAS-STING pathway and inflammation (Paul et al., 2021).

It was previously reported (Lv et al., 2022) that Tere^-/-^ aged mice were more sensitive to respiratory viral infections such as IAV and SARS-CoV-2, exhibiting excessive inflammatory responses, typical senescence features and increased mortality, which further abnormally activated the cGAS-STING pathway and NLRP3 inflammasome by a process that is mainly mediated by leaked mtDNA. Compared with normal controls, the mitochondria in Tere^-/-^ macrophages showed a swollen shape, irregular rarefied cristae and compromised ATP generation, as well as increased mROS stress. Consistently, there was an elevated amount of cytoplasmic mtDNA in Terc^−/−^ macrophages upon IAV infection, while less mtDNA was retained in mitochondria. Of note, the above phenotypes were more visible following viral infection. However, targeted inhibition of mtDNA release *via* VBIT-4 significantly weakened the abnormal activation of the cGAS-STING pathway in Tere^-/-^ macrophages relative to controls (Lv et al., 2022), suggesting the importance of aging-related mitochondrial dysfunction in response to viral infection in triggering exaggerated inflammatory responses and causing severe organ damage. Further effects of viral infection on mitochondrial function through the induction of more VIS may play a vital role in the higher levels of mtDNA liberation, leading to stronger inflammatory responses.

An increasing number of research showed that aberrant activation of the aging-associated cGAS-STING pathway and NLRP3 inflammasome underlies the increased lethality of SARS-CoV-2 infection in the elderly, and activation of the NLRP3 inflammasome may be mediated *via* the cGAS-STING pathway (Lara et al., 2020; Wang et al., 2020b; Lv et al., 2022). Specifically, telomere dysfunction in the elderly stimulates p53-mediated cellular responses and inhibits major regulators of mitochondrial function such as PGC-1α and PGC-1β, conducing to impaired mitochondrial function, enhanced oxidative stress and mtDNA accumulation. Higher levels of mtDNA can generate sustained activation of the cGAS-STING pathway and NLRP3 inflammasome, as well as elevated levels of pro-inflammatory factors. When viral infection occurs, VIS further boosts these pathways and facilitates more production of pro-inflammatory factors, inflicting greater damage to the organism (Kang et al., 2018; Lara et al., 2020; Lv et al., 2022). In addition to SARS-CoV-2 and IAV, when exposed to a series of RNA or DNA viruses or viral products such as human rhinovirus, dengue virus, adenovirus, HCV, MV, RSV, HIV and HSV, organisms demonstrate antiviral effects by activating inflammasomes, such as NLRP3 and AIM2 (Shrivastava et al., 2016), and PRRs, such as Toll-like receptors and RIG-I-like receptors, which are important for recognizing viruses in addition to cGAS (Kawai and Akira, 2008; Wilkins and Gale, 2010). Thereinto, the toll-like receptor-3 (TLR-3) proved to exacerbate SASP secretion of human senescent cells upon SARS-CoV-2 infection (Tripathi et al., 2021). The Toll-like receptor 2 (TLR2) and its partner TLR10 were shown to be key mediators of senescence *in vitro* and in murine models during oncogene-induced senescence (OIS). TLR2 can promote cell cycle arrest by regulating tumor suppressors p53-p21, p16 and p15 and modulate the SASP production by inducing acute-phase serum amyloids A1 and A2 (Hari et al., 2019). However, little is known about whether antiviral responses induced by other PRRs or inflammasomes are linked to aging or aging-related excessive inflammation and tissue damage.

Notably, RNA viruses such as IAV and SARS-CoV-2 are thought to be recognized by RNA receptors such as Toll-like receptors and RIG-I rather than cGAS, a DNA receptor, upon infections (Liu et al., 2016). In this regard, as previously described, for non-DNA virus infection, the infectious agent may indirectly trigger cGAS-STING activation by directly or indirectly inducing mitochondrial stress to leak mtDNA (Hanada et al., 2020; Hopfner and Hornung, 2020). Such a situation applies to the dengue virus and HSV. Dengue virus is a single positive-stranded RNA virus whose infection generates an endogenous source of cytoplasmic DNA through the release of mtDNA, which drives cGAS to produce cGAMP, with the latter subsequently binding and activating STING, which in return activate the NF-κB and IRF3 pathways and trigger an innate immune antiviral response (Ha et al., 2011; Aguirre et al., 2017; Sun et al., 2017). Although HSV is a DNA virus, its infection can also stimulate the liberation of mtDNA and activate the cGAS-STING pathway (West et al., 2015). Additionally, intracellular accumulation of retrotransposable elements can be reactivated during aging in somatic tissues to drive cGAS-dependent type I interferon responses and contribute to the maintenance of age-related inflammation (De Cecco et al., 2019). For example, HIV, a retrovirus, can trigger cGAS-STING reaction with its reverse-transcribed HIV DNA, and inhibitors of HIV reverse transcriptase can block the induction of interferon response by this virus (Gao et al., 2013). The binding of cGAS to HIV DNA is assisted by the host factor NONO, a multifunctional protein that binds nucleic acids and HIV capsid proteins in the nucleus. NONO is thought to directly recognize HIV DNA by nuclear-localized cGAS (Lahaye et al., 2018). In addition to NONO, host proteins such as PQBP1 (Yoh et al., 2015), ZCCHC3 (Lian et al., 2018) and G3BP1 (Liu et al., 2019) can also contribute to cGAS sensing of reverse-transcribed DNA.

#### 4.1.2 Pre-activated macrophages and over-recruitment of immune cells

It was found that compared to resting macrophages in the lungs of young mice, resident pulmonary macrophages from old mice were in an activated state and more likely to be activated in response to infections. Moreover, these aged lung macrophages harbored higher basal levels of circulating pro-inflammatory cytokines, such as IL-1β, IL-6 and TNF-α (Canan et al., 2014). Saskia L Smits et al. previously reported that SARS-CoV-infected aged macaques developed more severe pathology and higher lethality with a stronger host response than young adult animals, even though viral replication levels were similar and the mRNA levels of IFN-β were negatively correlated with gross pathology. However, treatment with type I interferon significantly diminished the expression of pro-inflammatory genes and attenuated the pathological response in old macaques (Smits et al., 2010). This could be explained by the role of pre-activated macrophages and higher basal levels of pro-inflammatory factors in aged individuals, and also to some extent by the fact that fatal viral infections in the elderly are often associated with exuberant inflammatory cell infiltration and delayed interferon production (Arunachalam et al., 2020).

In addition, previous studies showed that aging could increase mortality from influenza virus infection (Thompson et al., 2003). Senescent alveolar epithelial cells recruit excessive neutrophils (PMNs) in old mice by secreting higher levels of chemokines CXCL1 and CXCL2 upon influenza virus infection (Kulkarni et al., 2019). More importantly, activated PMNs can also generate more pro-inflammatory factors, further recruiting immune cells and leading to more severe inflammatory responses and tissue damage relative to young mice (Peiró et al., 2018; Kulkarni et al., 2019). However, the depletion of PMNs following viral infection can substantially improve the survival of aged mice without altering viral clearance (Kulkarni et al., 2019).

Collectively, the pre-existing inflammatory state and the over-recruitment of immune cells in response to viral infections in older individuals can precipitate increased inflammatory responses to external pathogens, resulting in a massive release of inflammatory mediators and potentially causing widespread tissue damage in common and non-fatal infections for the elderly.

#### 4.1.3 Role of immune cells with trained immunity

Immune memory is traditionally regarded as an exclusive hallmark of adaptive immunity. However, activation of the innate immune system can also lead to an enhanced response to secondary infections, termed “trained immunity”, which is actually a form of innate immune memory (Netea et al., 2020a). Maojun You et al. revealed the establishment of trained immunity in COVID-19 convalescent individuals *via* the single-cell epigenomic landscape of peripheral immune cells, showing that trained and activated states of CD14^+^ and CD16^+^ monocytes were dominantly enriched in individuals recovering from COVID-19 (You et al., 2021). These observations indicate that innate immune cells can form a non-specific but stable immune memory after initial infection, although it may be transient compared to classical T and B cells (Netea et al., 2020a; You et al., 2021). Furthermore, this epigenomic regulation of the innate immune memory response may not be specific to SARS-CoV-2 but also be elicited following other infections such as SARS-CoV-1, MERS, HIV, or vaccination, which is deemed to be a fundamental characteristic of host defense of multicellular organisms, including mammals (Netea et al., 2020a; You et al., 2021; Sviridov et al., 2022).

Evidence accumulated in recent years suggests that trained immunity caused by epigenetic and metabolic reprogramming is a double-edged sword. Although it enables a rapid and efficient host immune response to reinfected pathogens, it can also induce chronic inflammatory diseases (Netea et al., 2020a; Netea et al., 2020b; You et al., 2021). In the elderly, the accumulation of immune cells with trained immunity in the body may promote excessive inflammation and cause more severe tissue damage in the event of reinfection. Thus, appropriate targeting of immune cells with trained immunity in elderly individuals might be beneficial to relieve inflammation (You et al., 2021; Lv et al., 2022).

### 4.2 Aging-related multi-organ dysfunction

The effects of viral infections on the organism often involve multiple systems and organs. For example, SARS-CoV-2, a respiratory virus, in addition to causing lung infection, the virus can also replicate in cells of the intestine, liver and kidney, thus causing a variety of clinical symptoms other than the respiratory tract, such as gastrointestinal disorders, liver and kidney dysfunction (Chu et al., 2020) and even multi-organ failure (Chen et al., 2020). However, there is growing evidence supporting the role of aging in multi-organ dysfunction caused by viral infections (**Figure 2**).

Coagulation abnormalities and thrombosis can occur in the late stages in patients with viral infections and are often associated with poor prognosis (Nehme et al., 2020). Several factors, however, including aging, have been shown to be risk factors for vascular dysfunction (Nehme et al., 2020). On the one hand, senescent cells can secrete large amounts of SASP pro-inflammatory mediators that may trigger endothelial injury and favor thrombosis. On the other hand, together with inflammatory factors, senescent endothelial cells can shift the balance between pro- and anticoagulant pathways towards an elevated risk for thrombosis *via* the upregulation of factors that induce platelet aggregation such as plasminogen activator inhibitor-1 (PAI-1), thromboxane A2 (TXA2) and von Willebrand factor (vWF), while downregulating factors that inhibit platelet aggregation such as endothelial NO synthase (eNOS), prostacyclin and thrombomodulin (Bochenek et al., 2016; Wiley et al., 2019; Nehme et al., 2020). Therefore, the elderly may develop more severe coagulation disorders if infected by SARS-CoV, MERS-CoV, H1N1, HIV or other viruses (Obi et al., 2019; Ackermann et al., 2020; de Magalhães et al., 2020; Giannis et al., 2020). It was found that compared with young controls, aged hamsters exhibited prolongation of PT, intravascular clotting and acute kidney damage upon SARS-CoV-2 infection (Ohno et al., 2021). These changes are often present in patients with COVID-19 and strongly associated with disease severity and higher mortality (Helms et al., 2020; Porfidia et al., 2020; Loo et al., 2021). In addition, H1N1 acute respiratory distress syndrome (ARDS) patients possessed a 23.3-fold higher risk for pulmonary embolism and a 17.9-fold increased risk for venous thromboembolism (Obi et al., 2019). Simultaneously, in HIV-infected patients, endothelial dysfunction caused by HIV replication may also lead to a hypercoagulable state (Kuller et al., 2008; Armah et al., 2012), while aging-related inflammation and cellular changes may further contribute to coagulation dysfunction (de Magalhães et al., 2020).

Additionally, due to abnormal immune responses and excessive inflammation associated with aging, the elderly are more prone to complications such as liver and kidney dysfunction, myocardial injury, and neurological symptoms in the event of viral infections such as SARS-CoV-2. In particular, the massive secretion of SASP may generate fibrosis or cause injuries in organs other than the lungs, such as the liver, kidney and cardiovascular system (Cai et al., 2020; George et al., 2020; Napoli et al., 2020; Sharma et al., 2020; D'Agnillo et al., 2021). A decline in the blood-brain barrier function with aging may also cause infection of the central nervous system, leading to neurological symptoms (Yamazaki et al., 2016; Mao et al., 2020; Propson et al., 2021).

### 4.3 Dual role of aging antiviral response

Presently, it is believed that aging may cause dual effects during antiviral infection. Primarily, SASP cytokines and chemokines from senescent cells and accordingly recruited innate immune cells such as PMNs may predispose virus-induced senescence to become a part of the antiviral immune response (Baz-Martínez et al., 2016). This antiviral mechanism may enable the secretion of SASP factors by virus-induced senescent cells to restrict virus replication in neighboring cells and avoid its spread (Kelley et al., 2020). Moreover, the human papillomavirus (HPV), HBV, EBV and KSHV have evolved various mechanisms that can specifically combat cellular senescence (Yang et al., 2000; Oishi et al., 2007; Leidal et al., 2012; Zhi et al., 2015; Estêvão et al., 2019), indirectly suggesting that senescence may lead to antiviral defense in certain circumstances (Kelley et al., 2020).

Conversely, it was documented that senescence is conducive to the pathophysiology of viral infections and may promote viral replication and mutagenesis (Kelley et al., 2020; Evangelou et al., 2022). For instance, RSV infection can alter human airway epithelial differentiation and trigger the senescence of lung epithelial cells both *in vivo* and *in vitro* by generating ROS and causing DNA damage, thereby contributing to airway tissue remodeling and the severity and long-term consequences of RSV infections (Persson et al., 2014; Martínez et al., 2016). The influenza and varicella-zoster viruses can replicate more efficiently in senescent human bronchial epithelial cells and senescent human dermal fibroblasts, respectively, compared with non-senescent cells (Kim et al., 2016). The possible reasons for this phenomenon are the downregulation of type I interferon induction upon senescence and the defective mitochondrial dynamics of senescent cells, which consequently inhibit interferon expression and early interferon responses, thus favoring viral replication (Kim et al., 2016; Kelley et al., 2020). However, Baz-Martínez M et al. found that primary or chemotherapy-induced senescence reduces VSV replication (Baz-Martínez et al., 2016), suggesting that senescence plays a different role in response to diverse viral infections under distinguishing conditions. Remarkably, recent studies revealed that infected senescent cells might be a source of apolipoprotein B mRNA-editing (APOBEC) enzyme-mediated SARS-CoV-2 mutations (Evangelou et al., 2022).

**Figure 3:**
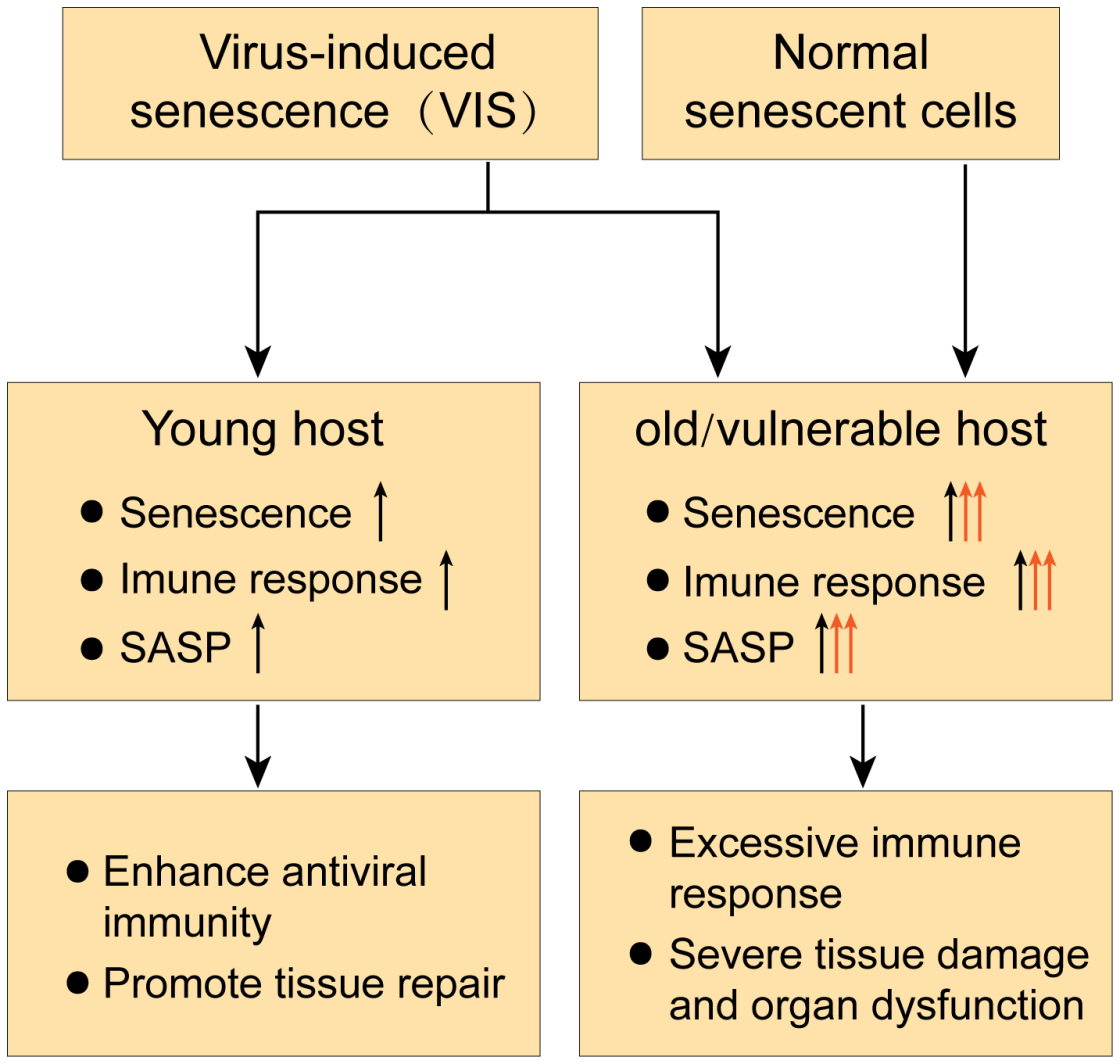
Dual role of senescence in antiviral immune responses.

Taken together, the effects of senescence on the body’s antiviral immune response are multifaceted (**Figure 3**). In this regard, it has been hypothesized from the perspective of acute respiratory viral infections that aging may play different roles in viral infections depending on host resilience (Kelley et al., 2020). In young hosts, VIS may enhance antiviral immunity by recruiting PMNs and other immune cells *via* SASP, thereby promoting viral clearance and tissue repair. However, in old or vulnerable hosts, VIS coupled with an existing senescence condition may lead to excessive immune responses with high levels of SASP cytokines and chemokines, resulting in secondary senescence and over-recruitment of immune cells, eliciting severe tissue damage and multi-organ failure (Kelley et al., 2020; Camell et al., 2021).

## 5 Significance of senotherapeutics in viral infectious diseases

In the presence of viral infections, VIS plus naturally occurring senescence are pivotal in precipitating excessive inflammatory responses, severe organ damage, and higher mortality in the elderly. Hence, senotherapeutics seem to be of great importance in alleviating clinical symptoms and organ damage and influencing disease regression in elderly individuals with viral infections.

Actually, for viral infectious diseases in aged people, there are currently two main areas of research in senotherapeutics (**Table 2**). The first one is the targeted removal of senescent cells, termed “senolytics”, mainly by propping up apoptosis of senescent cells, such as quercetin and fisetin (natural flavonoids), navitoclax (an inhibitor of BCL-2 pro-survival family) and dasatinib (a tyrosine kinase inhibitor). The second one is the inhibition of diverse components of SASP or inflammatory pathways involved in SASP synthesis, known as “senomorphics”, such as targeted inhibition of the cGAS-STING and NF-κB pathways or IL-1 and IL-6 cytokines. Intriguingly, available evidence implies a positive effect on mitigating aging-related diseases by eliminating senescent cells or inhibiting SASP secretion, and various clinical studies on senotherapeutics applied to viral infectious diseases are ongoing or have been completed (**Table 3**).

**Table 2:** Senescence-targeted therapeutics of viral infectious diseases.

Drugs	Main targets	Effect of treatment	Refs
Senolytics			
Quercetin	PI3K	Reduce the burden of senescent cells and major SASP factors, improving health	(Camell et al., 2021; Lee et al., 2021; Di Pierro et al., 2021a; Di Pierro et al., 2021b; Lv et al., 2022)
Fistein	PIK3/AKT	Alleviate cellular senescence features and mitochondrial damage, inhibit abnormal activation of cGAS-STING pathway and NLRP3 inflammasome	(Camell et al., 2021; Lee et al., 2021; Lv et al., 2022)
Dasatinib	Tyrosine kinases	Reduce the burden of senescent cells and major SASP factors, improving health	(Camell et al., 2021; Lee et al., 2021; Lv et al., 2022)
Navitoclax	Bcl-2, Bcl-xL, Bcl-w	Alleviate cellular senescence features and improve prognosis	(Chang et al., 2016; Lee et al., 2021)
Senomorphics			
Rapamycin	mTOR	Inhibit SASP generation, enhance antiviral activity and prolong healthy lifespan	(Wilkinson et al., 2012; Mannick et al., 2014; Wang et al., 2014; Herranz et al., 2015; Kindrachuk et al., 2015; Husain and Byrareddy, 2020)
Metformin	NF-κB	Inhibit the NF-κB pathway and pro-inflammatory factors production, resulting in a significant reduction in mortality	(Valencia et al., 2017; Crouse et al., 2020; Li et al., 2020; Luo et al., 2020; Xian et al., 2021)
Anakinra	IL-1R	Relieve clinical symptoms of viral infection and improve prognosis	(Cauchois et al., 2020; Huet et al., 2020)
Tocilizumab	IL-6R	Relieve clinical symptoms of viral infection and reduce mortality	(Group, 2021; Gupta et al., 2021)

**Table 3:** Clinical studies on senotherapeutics applied to viral infectious diseases.

Main targets	Agents	NCT Number	Conditions	Phase	Status	Dates	
						First Posted	Last Update Posted
PI3K NF-κB	Quercetin &Curcumin	NCT05601180 NCT05037240 NCT04861298 NCT04853199 NCT04578158 NCT04377789 NCT04851821 NCT01438320 NCT05130671	Long COVID COVID-19 COVID-19 COVID-19 COVID-19 COVID-19 COVID-19 Chronic Hepatitis C COVID-19	- - - I III - I I -	Recruiting Completed Completed Completed Completed Completed Completed Completed Completed	2022-11-01 2021-09-08 2021-04-27 2021-04-21 2020-10-08 2020-05-06 2021-04-20 2011-09-22 2021-11-23	2022-11-01 2021-09-08 2022-02-07 2023-01-04 2021-04-22 2021-02-18 2023-01-04 2015-03-20 2022-01-28
PI3K/AKT	Fistein	NCT04771611 NCT04537299 NCT04476953	COVID-19 COVID-19 COVID-19	II II II	Enrolling by invitation Enrolling by invitation Enrolling by invitation	2021-02-25 2020-09-03 2020-07-20	2023-01-18 2023-01-25 2023-01-25
TKI	Dasatinib	NCT05527418	HIV-1 Infection	II	Not yet recruiting	2022-09-02	2022-09-02
NF-κB TKI	Isoquercetin &Masitinib	NCT04536090 NCT04622865	COVID-19 COVID-19	II II	Not yet recruiting Recruiting	2020-09-02 2020-11-10	2021-08-23 2022-06-22
mTOR	Rapamycin RTB101	NCT04948203 NCT04461340 NCT04341675 NCT04584710 NCT04409327	COVID-19/Long COVID COVID-19 COVID-19 COVID-19 COVID-19	II/III II II II II	Recruiting Unknown Unknown Active, not recruiting Terminated	2021-07-01 2020-07-08 2020-04-10 2020-10-14 2020-06-01	2022-11-22 2020-09-09 2020-05-20 2021-02-09 2021-02-10
NF-κB	Metformin	NCT04625985	COVID-19	II	Completed	2020-11-12	2021-08-08
IL-1R IL-6R IFN-γ JAK JAK1/2 IL-6R JAK1/2 IL-6R IL-6	Anakinra &Tocilizumab &Emapalumab &Baricitinib &Ruxolitinib &Tocilizumab and Ruxolitinib &Tocilizumab and Siltuximab	NCT05611710 NCT04680949 NCT04643678 NCT04462757 NCT04443881 NCT04364009 NCT04362111 NCT04357366 NCT04341584 NCT04412291 NCT04339712 NCT04324021 NCT04362943 NCT04366232 NCT04424056 NCT04330638	Dengue COVID-19 COVID-19 COVID-19 COVID-19 COVID-19 COVID-19 COVID-19 COVID-19 COVID-19 COVID-19 COVID-19 COVID-19 COVID-19 COVID-19 COVID-19	II III II/III II II/III III III II II II II II/III - II III III	Not yet recruiting Completed Completed Terminated Completed Terminated Active, not recruiting Active, not recruiting Completed Unknown Completed Terminated Completed Terminated Unknown Completed	2022-11-10 2020-12-23 2020-11-25 2020-07-08 2020-06-23 2020-04-27 2020-04-24 2020-04-22 2020-04-10 2020-06-02 2020-04-09 2020-03-27 2020-04-27 2020-04-28 2020-06-09 2020-04-01	2022-11-10 2022-09-06 2022-08-16 2021-04-30 2021-06-01 2021-01-15 2023-01-26 2023-01-13 2021-02-01 2021-02-18 2021-01-11 2022-03-10 2021-07-28 2020-12-16 2020-06-23 2021-09-29
IL-6R JAK	Tocilizumab &Baricitinib	NCT05164133 NCT05057962 NCT04924829 NCT04893031 NCT04730323 NCT04479358 NCT04445272 NCT04412772 NCT04403685 NCT04377750 NCT04377659 NCT04372186 NCT04363853 NCT04363736 NCT04359667 NCT04356937 NCT04346355 NCT04335071 NCT04332913 NCT04332094 NCT04331795 NCT04320615 NCT04317092 NCT04315480 NCT05082714	COVID-19 COVID-19 COVID-19 COVID-19 COVID-19 COVID-19 COVID-19 COVID-19 COVID-19 COVID-19 COVID-19 COVID-19 COVID-19 COVID-19 COVID-19 COVID-19 COVID-19 COVID-19 COVID-19 COVID-19 COVID-19 COVID-19 COVID-19 COVID-19 COVID-19	I - - - IV II II III III IV II III II II - III II II - II II III II II -	Recruiting Completed Recruiting Completed Completed Recruiting Completed Unknown Terminated Unknown Terminated Active, not recruiting Unknown Completed Unknown Completed Terminated Terminated Unknown Recruiting Completed Completed Unknown Unknown Recruiting	2021-12-20 2021-09-27 2021-06-14 2021-05-19 2021-01-29 2020-07-21 2020-06-24 2020-06-02 2020-05-27 2020-05-06 2020-05-06 2020-05-01 2020-04-27 2020-04-27 2020-04-27 2020-04-22 2020-04-15 2020-04-06 2020-04-03 2020-04-02 2020-04-02 2020-03-25 2020-03-20 2020-03-19 2021-10-19	2023-01-18 2022-05-17 2021-06-14 2021-05-20 2021-01-29 2022-05-18 2021-06-02 2020-11-17 2020-08-26 2020-05-06 2022-11-02 2021-09-27 2020-11-30 2022-08-31 2020-11-12 2021-07-27 2020-06-22 2020-10-14 2020-04-13 2021-05-06 2022-06-09 2021-06-30 2021-03-03 2020-04-13 2022-04-13

PI3K, PhosphoInositide-3 Kinase; TKI, tyrosine kinase inhibitor; IL, interleukin; IL-1R, interleukin-1 receptor; IL-6R, interleukin-6 receptor; JAK, Janus kinase; mTOR, mammalian target of rapamycin; NF-κB, nuclear factor-κB.

### 5.1 Senolytics

The most widely studied therapeutic strategy for the targeted elimination of senescent cells by senolytics is the combination therapy of dasatinib (D) with quercetin (Q). “D+Q” treatment was shown to reduce the cellular stress of aged mice, lessen vascular sclerosis, strengthen vasodilatory functions and impel lung function in mice with pulmonary fibrosis, thus improving the health status and lifespan of elderly mice (Roos et al., 2016; Lehmann et al., 2017; Xu et al., 2018). Likewise, preliminary clinical trials have demonstrated that “D+Q” therapy significantly improved the physical function of elderly patients with pulmonary fibrosis, reduced the burden of senescent cells in diabetic patients with chronic kidney disease, decreased the levels of major circulating SASP factors, and slowed disease progression (Hickson et al., 2019; Justice et al., 2019).

In terms of viral infectious diseases, Camell et al. discovered that human endothelial senescent cells initiated excessive inflammation upon exposure to SARS-CoV-2, which was accompanied by enhanced SASP expression and a pronounced increase in cellular senescence, inflammation and mortality among aged mice with similar β-coronavirus infection (Camell et al., 2021). However, the use of fisetin or the “D+Q” therapy with senescence-targeted ability selectively combated senescent cells, substantially reduced the signs of senescence and the levels of inflammatory markers and decreased viral infections-related mortality (Camell et al., 2021). In a recent study (Lee et al., 2021), SARS-CoV-2 virus infection and the subsequent VIS were determined to be driving factors in modulating COVID-19-related cytokine storm and tissue damage. However, the targeted removal of senescent cells with senescence-targeted drugs such as navitoclax, D+Q and fisetin lead to a significant reduction in senescent cells and a marked attenuation of senescence-related traits, both *in vitro* and in the respiratory epithelium of hamster models. Further, COVID-19-related lung diseases, inflammation, tissue damage and coagulation disorders significantly subsided (Lee et al., 2021). Additionally, in senescent macrophages exposed to IAV or SARS-CoV-2 (Lv et al., 2022), fisetin prominently suppressed the aberrant activation of the cGAS-STING pathway, NLRP3 inflammasome and the resultant excessive inflammatory responses in senescent macrophages *via* the induction of apoptosis and reduction of dysfunctional mitochondrial load. In the Tere^-/-^ aged mice model, fisetin was also found to dampen pathogenic inflammation mediated by the cGAS-STING pathway and NLRP3 inflammasome by lowering senescent cells-related burden and improving mitochondrial integrity, thus providing a considerable improvement in the survival rate of Tere^-/-^ mice infected with IAV. Comparable results were obtained upon validation using the “D+Q” combination treatment (Lv et al., 2022).

In prospective randomized controlled clinical trials among patients with COVID-19, quercetin appeared to be effective in decreasing viral load and relieving clinical symptoms during the early application of viral infection in combination with standardized care, which offered protection against serious complications and conferred a high safety profile (Di Pierro et al., 2021a; Di Pierro et al., 2021b). Correspondingly, a systematic review of quercetin revealed that quercetin and its derivatives were associated with significantly reduced mean viral load and generation of pro-inflammatory cytokines, chemokines, reactive oxygen species, mucus and airway resistance in animals infected with respiratory viruses such as influenza virus and human rhinovirus. These observations were associated with a significant reduction in infected animal fatality and were considered a potential strategy for treating lower respiratory tract viral diseases (Brito et al., 2021).

### 5.2 Senomorphics

The mammalian target of rapamycin (mTOR) pathway has been demonstrated to facilitate SASP production in recent years by regulating the translation of mRNA subsets, including those encoding IL-1α (Herranz et al., 2015; Laberge et al., 2015). Rapamycin can target the mTOR pathway to inhibit the activity of the mTORC1 complex, which is known to regulate mRNA translation, leading to reduced mRNA levels of cytokines such as IL-6 and IL-10 and selective inhibition of the translation of IL-1α. This creates a drop in SASP production and reduces the risk of age-related cognitive decline and cardiac or hepatic dysfunction, eventually extending the lifespan of mice and improving immune functions in the elderly (Majumder et al., 2012; Wilkinson et al., 2012; Flynn et al., 2013; Mannick et al., 2014; Laberge et al., 2015). More importantly, the application of rapamycin can potentiate antiviral activity in the event of SARS-CoV-2, MERS-CoV, H1N1 and other viral infections, which can be conducive to attenuating the severity of diseases (Wang et al., 2014; Kindrachuk et al., 2015; Husain and Byrareddy, 2020). Currently, ongoing clinical trials are assessing the safety and efficacy of rapamycin in the treatment or prevention of COVID-19 (Geier and Perl, 2021).

In addition, metformin, a biguanide that combats age-related diseases to extend health span, is the first drug for age-targeted effects in a large clinical trial (Kulkarni et al., 2020). Available evidence supports that metformin can dampen aging-related features directly or indirectly through multiple pathways, such as improving nutrient perception, inhibiting the NF-κB pathway and pro-inflammatory factors production, enhancing cellular autophagy and intercellular communication, regulating mitochondrial function, modulating gut microbiota, delaying stem cell aging, curtailing telomere attrition, and attenuating cellular senescence (Kulkarni et al., 2020). It was reported that metformin could extend the lifespan of mice by inhibiting the production of pro-inflammatory factors and diminishing DNA damage (Martin-Montalvo et al., 2013; Moiseeva et al., 2013; Valencia et al., 2017). It could also decrease age-related chronic inflammation and the risk of cardiovascular diseases, cancer, neurodegenerative diseases and cognitive dysfunction, ultimately exerting a constructive effect on improving the overall health status and prolonging the lifespan of aged persons (Barzilai et al., 2016; Campbell et al., 2017; Markowicz-Piasecka et al., 2017; Campbell et al., 2018; Tizazu et al., 2019).

Data from China revealed that the metformin treatment was associated with lower mortality in hospital patients with COVID-19 (Li et al., 2020; Luo et al., 2020) and fewer COVID-19-related heart failure and inflammation compared with other anti-diabetic agents (Cheng et al., 2020). Similarly, metformin could also cause an almost 11-fold reduction in the odds ratio of death among COVID-19 African-American patients with T2DM (Crouse et al., 2020). A recent study confirmed that metformin inhibited mtDNA synthesis and cytoplasmic Ox-mtDNA production in macrophages, thereby suppressing NLRP3 inflammasome activation, IL-1β generation and IL-6 secretion and relieving lung inflammation in human ACE2 transgenic mice infected with SARS-CoV-2 (Xian et al., 2021). Such protective effects were independent of glycemic control and correlated with the anti-inflammatory properties of metformin (Valencia et al., 2017; Marcucci et al., 2020). Nevertheless, it should also be noted that metformin use is linked to a high incidence of acidosis, especially in severe COVID-19 cases (Cheng et al., 2020), and careful consideration should be made on tackling the complications during clinical administration.

Anakinra, an IL-1 receptor antagonist, was shown to cause a rapid decrease in inflammatory and febrile symptoms, lower oxygen requirements, increase the duration of non-invasive mechanical ventilation and improve various clinical conditions when administered early in COVID-19 patients (Cauchois et al., 2020). In parallel, another cohort study also concluded that therapy with Anakinra was associated with fewer needs for invasive mechanical ventilation, lowered the mortality of patients with severe COVID-19, and, importantly, did not cause serious side effects (Huet et al., 2020). Moreover, a multicenter cohort study enrolling 3924 COVID-19 patients suggested that treatment with a monoclonal antibody of the IL-6 receptor (Tocilizumab) during the first 2 days of patient admission to the ICU could significantly reduce the risk of in-hospital mortality (Gupta et al., 2021). A randomized controlled clinical trial from the United Kingdom also demonstrated that Tocilizumab lowered the probability of invasive mechanical ventilation needs and 28-day mortality in patients with COVID-19 (Group, 2021). Further, a Bruton tyrosine kinase (BTK) inhibitor was also found to reduce BTK-dependent activation of NF-κB and NLRP3 inflammasome, which suppressed pro-inflammatory factors production and COVID-19 cytokine storm, thereby improving the prognosis of COVID-19 patients (Roschewski et al., 2020).

Altogether, these findings not only further illustrate the critical role of SASP components in the development of viral infectious diseases such as COVID-19 but also demonstrate the high interest in targeting SASP components or the inflammatory pathways involved in their synthesis for the treatment of aging-related viral infectious diseases.

## 6 Conclusion and outlook

Senescence and viral infections interact in a reciprocal relationship. In general, viral infections can induce senescence and increase the susceptibility and severity of viral infections *via* multiple mechanisms, such as immunodeficiency, mitochondrial dysfunction, SASP secretion, pre-activated macrophages, over-recruitment of immune cells, and accumulation of innate immune cells with trained immunity. In the elderly, virus-induced senescence, in addition to their pre-existing senescent condition, is believed to aggravate the underlying disease outcomes, but could be counteracted by senotherapeutics, which was shown to mitigate the severity of viral infections.

Undeniably, well-controlled senescence onset may positively enhance antiviral immunity, yet excessive inflammatory responses by accumulated senescent cells are critical factors underlying the development of multiple aging-related diseases. However, the relationship between viral infections and senescence should be further clarified, because it remains undetermined whether the effects are fully compatible between virus-induced senescence and naturally occurred senescence on the antiviral responses of hosts, the exact mechanisms of virus-induced senescence are not fully clear, the optimal doses of anti-senescence therapeutic drugs remain investigational, and the specific adverse events are not yet fully known. Thus, further research and clinical trials are needed to prolong a healthy lifespan of the elderly.

## Author contributions

ZL, MT, and CZ conceptualized and designed this study. ZL, MT, and GW wrote the original draft and prepared the diagrams. XC, J’eM, and SL researched data and collected the references. XC and BS reviewed and edited the manuscript. CZ, XX, KW, and FL critically revised the manuscript. All authors contributed to the article and approved the submitted version.
